# Clinical and Epidemiological Study of Leptospirosis in the Settlements of Presidente Epitacio and Mirante do Paranapanema

**DOI:** 10.3390/microorganisms13040865

**Published:** 2025-04-10

**Authors:** Mario A. R. Aleman, Natalia C. Gaeta, Vanessa Castro, Eduardo C. Marques, Bruno L. M. Ribeiro, Zenaide S. Olímpio, Lilian Gregory

**Affiliations:** 1 University of Valle de Puebla, Puebla 72440, Mexico; mvz_ram@live.com.mx; 2 School of Veterinary Medicine and Animal Science, University of São Paulo, São Paulo 05508-220, Brazil; natalia.gaeta@hotmail.com (N.C.G.); eduardovetusp@gmail.com (E.C.M.); zenaide_sous@usp.br (Z.S.O.); 3 Biological Institute of the State of São Paulo, São Paulo 04016-035, Brazil; vanessa.castro@sp.gov.br; 4 Department of Internal Medicine, School of Veterinary Medicine, Federal University of Mato Grosso, Sinop 78550-728, Brazil; brunolmribeiro@gmail.com

**Keywords:** *Leptospira* spp., bovine clinic, reproductive diseases

## Abstract

Brazilian family farming comprises production units that represent approximately 86.40% of agricultural establishments, playing a significant national role in socio-environmental and economic sectors. The state of São Paulo is known for its rural and urban development, including agricultural settlements such as Pontal do Paranapanema. This sector requires attention due to the emergence of infectious diseases, such as leptospirosis, a widespread zoonotic disease that causes losses in agricultural productivity. This study aimed to analyze the prevalence of leptospirosis in dairy cattle within the settlements of Presidente Epitácio and Mirante do Paranapanema. All animals underwent serological testing. The overall prevalence of *Leptospira* spp. was 58.93%. Additionally, the prevalence of antibodies against *Leptospira* spp. was 59.20% in multiparous cows and 58.60% in primiparous cows. A correlation was also observed between animals with retained placenta and seropositive animals for *Leptospira* spp. antibodies. Based on this study, we highlight that the high prevalence of leptospirosis underscores the presence of this pathology in settlements within the Pontal do Paranapanema region. Consequently, there is a need to develop public policy programs, alongside sanitary and control measures, to mitigate the impact on dairy farming in the region.

## 1. Introduction

In Brazil, family farming has played an essential role in the country’s economy in recent years through its development and productivity contributions. This sector is responsible for productive units that account for approximately 86.40% of all agricultural establishments, contributing roughly 10% of the national GDP [1,2], thus fostering a socio-environmental, cultural, and economic legacy.

Compared to other regions in Brazil, São Paulo is recognized as a state with well-developed rural and urban areas, characterized by equitable and cost-effective agricultural settlements [3]. Among these regions is Pontal do Paranapanema, where dairy production stands out as a contributing factor to the region’s economic and commercial strength. According to information from the Secretariat of Economic Development, Pontal comprises 140 state settlements and 113 federal settlements [4].

This study was conducted in Pontal do Paranapanema, a region with 32 municipalities located in the far west of São Paulo at an altitude of 448 m [5]. This region is bordered by the Paraná and Paranapanema rivers and shares boundaries with the states of Mato Grosso do Sul and Paraná [6].

The climate in the Pontal do Paranapanema region is categorized as Cwa and Aw, according to the Köppen and Geiger classification. The Cwa climate is predominant and characterized by a mesothermal dry winter, with rainfall during summer and a dry season in winter, with average annual temperatures below 22 °C. In contrast, the Aw climate is a humid tropical climate, with a rainy summer and dry winter, average temperatures between 22 °C and 24 °C, and an average annual precipitation of 1500 mm [7].

The Pontal do Paranapanema region, primarily agricultural, also has the highest concentration of settlement communities, with a total of 6060 settled families [4]. This region has the lowest average per capita income in São Paulo, highlighting low socioeconomic status and vulnerability among the general population.

In Pontal do Paranapanema, approximately 500 L of milk are produced per producer each month [8]. This sector requires further development to improve these figures. Therefore, it is essential to emphasize that improving dairy production efficiency depends on a complex array of factors that need to be addressed comprehensively, including genetic factors, adequate nutrition and management, as well as the sanitary and environmental conditions of the animals. These elements directly impact animal and human health and contribute to the emergence of vectors and infectious diseases, such as leptospirosis, a disease of veterinary, zootechnical, and public health importance.

Leptospirosis is a zoonotic and cosmopolitan disease that affects domestic, wild, and synanthropic animals and, consequently, humans. The disease is caused by a bacterium of the genus *Leptospira* spp., belonging to the order Spirochaetales [7]. The main clinical signs include productive and reproductive issues, mammary gland afflictions, abortions, stillbirths, weak fetuses, and infertility, as well as the development of clinical or subclinical mastitis [9,10]. When mammary glands are affected, observable changes may include the presence of blood in the milk and/or a decrease in milk production [11,12].

The mechanism of action of *Leptospira* is complex, affecting both the innate and acquired immune systems [13,14]. Species of *Leptospira* are classified by pathogenicity into saprophytic, intermediate, and pathogenic types. Currently, at least 300 identified serovars are categorized into 28 serogroups [10,15,16,17].

In the agricultural sector, the most affected industry is cattle farming, involving both dairy and beef production [17]. Due to the epidemiological complexity of leptospirosis, Martins and Lilenbaum [18] report that effective control measures include antibiotic therapy, environmental control, and vaccination protocols. Currently, multivalent vaccines, composed of inactivated strains or bacterins from various *Leptospira* serovars, stimulate the animal’s immune system by enhancing the Th1 and Humoral response [19,20]. However, diagnostic errors often arise due to similarities between vaccine-induced responses and infections of *Leptospira* spp. in the herd, leading to inconclusive data. Therefore, it is important to improve studies for new vaccine development and enhance understanding of existing serovars [21,22].

According to the World Organization for Animal Health (2021) [23], the most common laboratory serological test is the microscopic agglutination test (MAT). However, this test has limitations, as it cannot identify specific genotypes and can produce cross-reactions among *Leptospira* species with similar antigenic characteristics, complicating serovar recognition and making precise diagnosis difficult [21,22,24]. Therefore, further studies on *Leptospira* are needed, focusing on correlating its distribution, prevalence, and clinical manifestations in animals.

The main objective of this study was to determine the prevalence of antibodies against *Leptospira* spp. in cows raised in the settlements of Presidente Epitácio and Mirante de Paranapanema in São Paulo. Additionally, this study evaluated the association of these microorganisms with reproductive disorders, management practices, and facilities available on the properties.

## 2. Materials and Methods

### 2.1. Characterization of the Study Area

This study was conducted in settlements within the territories of Presidente Epitácio and Mirante de Paranapanema, located in the Pontal do Paranapanema region, an agriculturally intensive area in which Presidente Prudente serves as the primary municipality. This region is situated in the southwestern part of São Paulo state, bordering the states of Mato Grosso do Sul and Paraná, and is geographically delineated by the Paraná and Paranapanema rivers [4].

### 2.2. Sampling

A two-stage sampling design was implemented to determine the required sample size of cows. The first stage involved selecting the properties to be visited, while the second stage involved sampling animals from each selected property. This process was facilitated using the EpiTool Epidemiological Calculator (SERG) from Ausvet (Fremantle, Australia). Parameters for the diagnostic test performance were set with a minimum sensitivity and specificity of 70%. An expected prevalence of at least 47% in animals and 10% in properties was assumed, with a 95% confidence level.

In total, 42 properties were visited, with proportional representation from the regions of Presidente Epitácio and Mirante de Paranapanema within Pontal do Paranapanema. Samples were collected from four animals per property. Specifically, in Presidente Epitácio, 12 properties were sampled, resulting in 48 animal samples. In Mirante de Paranapanema, 30 properties were visited, yielding 120 samples. This produced a total of 168 animal samples for analysis, as summarized in Table 1. Notably, none of the animals included in this study were vaccinated against leptospirosis.

### 2.3. Property Assessments and Clinical Examinations of Females

Evaluations of the visited properties were conducted using a standardized epidemiological questionnaire [25] administered through an interactive approach with rural property owners to gather comprehensive data on local conditions. The questionnaire covered property characteristics, historical and clinical assessments, reproductive factors, feeding practices, and observable clinical signs. The average herd size was 20 animals per farm.

To obtain specific information on the reproductive health of the females, a thorough examination of the reproductive system was performed to assess the association between seropositive or seronegative status and any existing reproductive manifestations. The overall condition of the animals, as well as their reproductive systems, was assessed following the methodologies outlined, with all findings systematically recorded in clinical records for subsequent analysis.

### 2.4. Samples

#### 2.4.1. Serum

Blood samples were collected in 2016 via venipuncture, drawing 8 mL from the jugular vein using vacuum system tubes BD Vacutainer^®^ dry (Becton, Dickinson and Company (BD), Franklin Lakes, NJ, USA). The tubes were subsequently centrifuged at 600× *g* for 15 min to isolate the serum, which was then transferred to microtubes and frozen for preservation. Samples were stored at the Laboratory of Reproductive Bacterial Diseases at the Biological Institute (IB) of the State of São Paulo until further analysis. In 2024, the obtained data were analyzed as part of this study, which holds significant relevance given the absence of research on the epidemiological prevalence of *Leptospira* spp. in Pontal do Paranapanema since 2016.

#### 2.4.2. Microscopic Agglutination Test for *Leptospira* spp.

To detect the presence of antibodies in the cattle, the microscopic agglutination test (MAT) was employed, recognized as the gold standard method for this purpose. This assay detects antibodies against *Leptospira* spp. and its serovars [26,27], with results observed under a microscope equipped with a dark-field condenser [28]. Twenty-two antigens obtained from the Laboratory of Bacterial Diseases were used, representing the following *Leptospira* serovars: Icterohemorrhagiae, Canicola, Pomona, Grippotyphosa, Wolffi, Hardjo, Australis, Autumnalis, Batavitae, Bratislava, Butembo, Castellonis, Copenhageni, Cynopteri, Hebdomadis, Javanica, Panama, Pyrogenes, Shermani, Tarassovi, Whitcombi, and Sentot. *Leptospira* cultures were maintained in specialized media to support their growth and viability, and a cutoff titer of 1:100 was established for positive results.

### 2.5. Statistics

Data analysis was conducted using SAS software, version 9.3 (SAS Institute, Cary, NC, USA). Variables were grouped to enhance the statistical significance of the data, with variable groups defined for reproductive disorders (including abortion, malformation, stillbirth, and dystocia) and vaginal abnormalities (such as discharge, vesicles, nodules, and pustules). A property was classified as positive if it contained at least one reactive animal or one with a positive serological result for the infectious agents under investigation. The PROC FREQ procedure was utilized to calculate the frequency of each variable, followed by the application of the chi-square test to identify significant differences in the percentages of the studied variables.

For cases where the number of experimental units was equal to or less than 15 k (where k represents the number of responses studied), the chi-square test was applied. When the frequency was below 20, or when the total frequency ranged between 20 and 40 with an expected frequency of less than 5, Fisher’s exact test was employed. A significance threshold of 5% (*p* < 0.05) was adopted for all statistical analyses.

## 3. Results

### 3.1. Microscopic Agglutination Test for Leptospira spp.

The results indicated that 58.93% (99/168) of the animals tested positive for *Leptospira* spp. (Table 2). Among positive samples, the highest prevalence of antibodies was observed for the following serovars: Wolfii (61.62%, 61/99), Hardjo (53.54%, 53/99), and Icterohemorrhagiae (51.52%, 51/99).

### 3.2. Relation Between Seroreactive Animals and Their Characteristics, Farms, and Reproductive Aspects

Table 3 below details the relation between seroreactive dairy cattle for *Leptospira* spp. and the characteristics observed in the animals and farms in settlements of Presidente Epitácio and Mirante do Paranapanema, São Paulo, Brazil (2016), along with their levels of significance (Table 4, Table 5, Table 6 and Table 7).

There was a significant relation between the presence of antibodies against *Leptospira* spp. and retained placenta (*p* = 0.0373), with an odds ratio of 0.574 (0.503–0.655).

On the other hand, there was no significance between the presence of antibodies against *Leptospira* spp. and the number of births (*p* = 1), body condition score (*p* = 0.1594), pregnancy (*p* = 0.1198), date of the last birth (*p* = 0.2819), reproductive alterations (*p* = 0.2264), and vaginal alterations (*p* = 0.3872).

Regarding the characteristics of the farms, there was also no significance between the presence of antibodies against *Leptospira* spp. and farm size (*p* = 0.5701), type of breeding system (*p* = 0.8606), type of feeding (*p* = 0.7348), and the presence of flooded areas (*p* = 0.8606).

Another factor that was evaluated and found to be not significant was the relation between the presence of antibodies against *Leptospira* spp. and the total number of animals (*p* = 0.3426), number of cows (*p* = 0.3916), number of calves (*p* = 0.4321), and reproductive management (*p* = 0.3909). When evaluating the presence of antibodies against *Leptospira* spp. and other domestic species (*p* = 0.7348), the results were also not significant. There was also no significance for the presence of antibodies against *Leptospira* spp. and abortion (*p* = 0.247), repeat breeding (*p* = 0.461), and stillbirths (*p* = 0.4187).

## 4. Discussion

Dairy cattle herds may harbor various infectious agents, such as *Leptospira*, which can lead to significant productivity and reproductive issues. Infected herds commonly experience conditions such as mastitis, flaccid udders, blood clots, and hemorrhages, resulting in milk production losses of up to 80%. Additional reproductive complications associated with *Leptospira* infection include abortions, fetal mummification or mortality, retained placenta, embryonic resorption, premature births, stillbirths, jaundice, and mortality [9,23]. Similarly, Leal [26] reported that retained placenta is a common and multifactorial issue affecting dairy herd productivity during the peripartum period. This condition can arise from factors such as excessive uterine distension during pregnancy, dystocia, premature births, poor or negligent management practices, stress, deficiencies in vitamins and minerals, hormonal imbalances, and infections like leptospirosis. In this context, Rocha [29] emphasized that the primary reproductive failures observed included abortions, retained placenta, and weak fetuses, which were the most frequently reported clinical manifestations. According to Sartori [30], the incidence of retained placenta is approximately 7.20% in primiparous cows and 12.20% in multiparous cows.

In this context, Rocha [29] observed that pregnant cows exhibit a greater susceptibility to acute symptoms compared to non-pregnant cows. Furthermore, pathogens such as *Leptospira* spp. display a pronounced tropism for uterine and placental tissues, leading to inflammatory responses [31] and lesions within the genital tract [32]. The present study corroborates these findings, revealing a significant association between the presence of antibodies against *Leptospira* spp. and the incidence of retained placenta in the animals studied.

In terms of prevalence, this study identified a seropositivity rate of 58.93% for *Leptospira* spp. Barnabé [33]; in similar studies conducted in the Brazilian states of Paraíba and Pernambuco, a seroprevalence rate of 64.30% has been reported. Likewise, in the Huertar Zone of northern Costa Rica, Soto [34] documented a positivity rate of 49.30%. These findings are consistent with prior research on leptospirosis, a globally prevalent disease in cattle, particularly within semi-intensive farming systems such as those evaluated in this study. These systems facilitate transmission through direct contact among animals, contributing to higher infection rates.

Among the serovars analyzed in this study, the most prevalent were Wolffi, Hardjo, and Icterohaemorrhagiae. In a related study, Gonçalves et al. [35] investigated the presence of *Leptospira* spp. DNA and antibodies in a Brazilian region bordering Paraguay. Using the microscopic agglutination test (MAT) and PCR, they analyzed 70 blood and vaginal mucus samples from female dairy cattle. Their findings indicated that 42.86% of the samples were reactive in MAT, with 43.47% of these reactive samples testing positive for the Wolffi serovar. Furthermore, *Leptospira* spp. DNA was detected in 74.28% of the vaginal mucus samples, suggesting that female cattle may act as potential carriers of this pathogen.

In Rio de Janeiro, Di Azevedo [36] analyzed 42 cows using blood samples and uterine fragments through serological and molecular techniques. The results from the MAT indicated that 20.50% of the animals were reactive to the Sejroe serogroup, while PCR targeting the “lipL32” gene revealed a 26.20% positivity rate for *Leptospira* spp. Sequencing of these samples identified nine strains, all characterized as *L. interrogans*, which were grouped within the Sejroe and Hardjo serogroups. In a related study, Souza [37] reported a higher prevalence of Hardjo (54.54%), Icterohaemorrhagiae (27.27%), and Autumnalis (18.18%) in cattle from farms in Uberlândia, Minas Gerais. Similarly, research by Santana et al. (2013) [38] in São Carlos, São Paulo, identified higher prevalence rates for the Pomona (63.30%), Hardjo (45.50%), Tarassovi (27.30%), and Wolffi (9.10%) serovars. The present study conducted in Presidente Epitácio and Mirante de Paranapanema is consistent with these findings, with the Wolffi, Hardjo, and Icterohaemorrhagiae serogroups showing the highest prevalence.

Aymeé [20] identified cattle as maintenance hosts, particularly for the Hardjo serovar (Sejroe serogroup), which includes the Hardjobovis and Hardjoprajitno serovars. Infected cows often develop chronic infections, leading to fertility disorders and reduced milk production. The findings of the present study align with these observations, corroborating the literature that designates this serovar as the most prevalent in cattle.

In this study, the presence of antibodies against *Leptospira* spp. was higher in multiparous cows (59.20%) compared to primiparous cows (58.60%). These results are consistent with those reported by Del Fava [39], suggesting that the increased exposure risk in older animals may account for the higher seropositivity rates observed.

No association was identified between the presence of *Leptospira* spp. and pregnancy status, consistent with the findings of Del Fava [39], who reported that the prevalence of antibodies against *Leptospira* spp. does not impact pregnancy outcomes.

In this study, 82.05% of the seropositive farms were 12 hectares or smaller, and 94.87% operated under semi-intensive or intensive farming systems, a pattern reflective of smaller farms, particularly in settlement areas, where these production systems are commonly adopted. However, Niang [40] noted that the prevalence of *Leptospira* spp. on a farm is largely attributable to the presence of carrier animals, which disseminate the pathogen into the environment through their urine. This contamination increases the risk of pathogen transmission among healthy animals, especially on small farms.

*Leptospira* spp. can persist in the environment for extended periods, with survival influenced by environmental factors such as humidity, temperature, and shade [39]. Feeding practices also affect the pathogen’s shedding through urine. Leonard et al. [41] observed that animals supplemented with grain silage exhibited lower urine pH, which over time reduced the *Leptospira* spp. concentrations in the urine.

In the current study, 56.40% of the positive properties housed more than 50 animals. Research by Martins [42] indicated that farms with over 21 animals present an increased risk for seroreactivity to any *Leptospira* spp. serovar. Similarly, Hashimoto et al. [43] found that in the south-central region of Brazil, farms with 43 or more females were more likely to test positive for *Leptospira* spp.

Natural breeding practices were observed on 82.10% of the seropositive farms. According to Martins [42], bulls frequently interact with wild animals, which serve as natural reservoirs for certain *Leptospira* spp. serovars. These wildlife reservoirs include various species such as small rodents, capybaras, coatis, and bats [44,45,46,47].

Another notable finding was that 89.74% of the seropositive farms housed other domestic species. Gerritsen et al. [48] reported that the presence of animals such as pigs, dogs, horses, and wild cervids significantly increases the likelihood of detecting the Hardjo serovar, as these species are recognized hosts of *Leptospira* spp.

## 5. Conclusions

This study, conducted in settlements within the Pontal do Paranapanema region (Mirante do Paranapanema and Presidente Epitácio), identified a *Leptospira* spp. prevalence of 58.90% among sampled animals. Significant correlations were observed between multiparous cows and the presence of *Leptospira* spp., as well as between retained placenta cases and seropositivity for *Leptospira* spp. antibodies.

Given the high prevalence of *Leptospira* spp. in these herds, it is recommended that the Pontal do Paranapanema region undergo monitoring through public health policies and the implementation of sanitary and control measures to reduce the pathogen’s impact on the dairy industry.

## Figures and Tables

**Table 1:** Distribution of properties visited and collected samples in the municipalities of Presidente Epitácio and Mirante de Paranapanema—São Paulo, Brazil, 2024.

Municipality (Pontal Paranapanema)	No. of Visited Properties N (%)	No. of Samples N (%)
Presidente Epitácio	12 (28.57)	48 (28.57)
Mirante de Paranapanema	30 (71.43)	120 (71.43)
Total	42 (100)	168 (100)

**Table 2:** Characterization of reactive and non-reactive samples in the microscopic agglutination test for detecting antibodies against *Leptospira* spp. in serum samples from cattle on farms in Presidente Epitácio and Mirante do Paranapanema, São Paulo, Brazil, 2016.

Serovars	Reactive % (N/Total)	Non-Reactive % (N/Total)
*Leptospira* spp.	58.90 (99/168)	41.07 (69/168)
*L*.Wolffi	61.60 (61/99)	
*L.* Hardjo	53.50 (53/99)	
*L.* Icterohaemorrhagiae	51.50 (51/99)	
*L.* Pomona	10.10 (10/99)	
*L.* Bratislava	9.10 (9/99)	
*L.* Pyrogenes	6.10 (6/99)	
*L.* Australis	1.10 (1/99)	

**Table 3:** Relation between the presence or absence of antibodies against *Leptospira* spp. according to female characteristics.

Female Characteristics			
Characteristics	Positive% (N/Total)	Negative% (N/Total)	*p*-Value
No. of calving • Primiparous • Multiparous	58.60 (41/99) 59.20 (58/99)	41.40 (29/69) 40.80 (40/69)	1.0000
Body condition score • ≤2.5 • 2.75 a 3.5 • 3.75≥	13.10 (13/99) 63.60 (63/99) 23.20 (23/99)	14.50 (10/69) 73.90 (51/69) 11.60 (8/69)	0.1594
Pregnancy • Pregnant • Non-pregnant	34.30 (34/99) 65.70 (65/99)	23.20 (16/69) 76.80 (53/69)	0.1198
Last calving • ≤3 months • 3 to 9 months • 10≥ months	31.30 (27/99) 48.50 (34/99) 20.2 (20/99)	39.10 (27/69) 49.30 (34/69) 11.60 (8/69)	0.2819
Reproductive alterations • Present • Absent	13.10 (13/99) 86.90 (86/99)	07.30 (5/69) 92.80 (64/69)	0.2264
Retained placenta • Present • Absent	06.10 (6/99) 93.90 (93/99)	0 (0/69) 100 (69/69)	0.0373
Vaginal alterations • Present • Absent	10.10 (10/99) 89.90 (89/99)	14.50 (10/69) 85.50 (56/69)	0.3872

**Table 4:** Relation between the presence or absence of antibodies against *Leptospira* spp. according to farm characteristics.

Farm Characteristics			
Characteristics	Positive% (N/Total)	Negative% (N/Total)	*p*-Value
Size • ≤12 hectares • >13 hectares	82.10 (32/39) 18 (7/39)	100 (3/3) 0 (0/3)	0.5701
System • Semi-intensive • Extensive	05.10 (2/39) 94.90 (37/39)	0 (0/3) 100 (3/3)	0.8606
Presence of flooded areas • Present • Absent	05.10 (2/39) 94.90 (37/39)	0 (0/3) 100 (3/3)	0.8606
Feeding type • Pasture • Silage	89.70 (35/39) 10.30 (4/39)	100 (3/3) 0 (0/3)	0.7348

**Table 5:** Relation between the presence or absence of antibodies against *Leptospira* spp. according to the number of animals.

No. of Animals			
Characteristics	Positive% (N/Total)	Negative% (N/Total)	*p*-Value
Total No. of animals • ≤50 animals • >50 animals	43.60 (17/39) 56.40 (22/39)	66.70 (2/3) 33.30 (1/3)	0.3426
No. of cows • ≤40 cows • >40 cows	71.80 (28/39) 28.20 (11/39)	100 (3/3) 0 (0/3)	0.3916
No. of calves • ≤25 calves • >25 calves	76.90 (30/39) 23.10 (9/39)	66.70 (2/3) 33.30 (1/3)	0.4321
Reproductive management • Artificial insemination • Natural breeding	18 (7/39) 82.10 (32/39)	33.30 (1/3) 66.70 (2/3)	0.3909

**Table 6:** Relation between the presence or absence of antibodies against *Leptospira* spp. according to contact with domestic animals.

Contact with Domestic Species			
Characteristics	Positive% (N/Total)	Negative% (N/Total)	*p*-Value
• Present • Absent	89.70 (35/39) 10.30 (4/39)	100 (3/3) 0 (0/3)	0.7348
Horses • Present • Absent	71.80 (28/39) 28.20 (11/39)	100 (3/3) 0 (0/3)	0.3916
Dogs • Present • Absent	56.40 (22/39) 43.50 (17/39)	100 (3/3) 0 (0/3)	0.2003
Cats • Present • Absent	07.70 (3/39) 92.30 (36/39)	0 (0/3) 100 (3/3)	0.7961
Domestic birds • Present • Absent	23.10 (9/39) 79.50 (31/39)	33.30 (1/3) 66.70 (2/3)	0.4321
Swines • Present • Absent	20.50 (8/39) 79.50 (31/39)	0 (0/3) 100 (3/3)	0.5213
Goats • Present • Absent	02.60 (1/39) 97.40 (38/39)	0 (0/3) 100 (3/3)	0.9286
Sheep • Present • Absent	23.10 (9/39) 79.50 (31/39)	0 (0/3) 100 (3/3)	0.4753

**Table 7:** Relation between the presence or absence of antibodies against *Leptospira* spp. according to reproductive alterations.

Reproductive Alterations			
Characteristics	Positive% (N/Total)	Negative% (N/Total)	*p*-Value
Abortions • Present • Absent	66.70 (26/39) 33.30 (13/39)	33.30 (1/3) 66.70 (2/3)	0.247
Repeated estrus • Present • Absent	33.30 (13/39) 66.70 (26/39)	33.30 (1/3) 66.70 (2/3)	0.461
Stillborns • Present • Absent	53.90 (11/39) 46.20 (18/39)	66.70 (2/3) 33.30 (1/3)	0.4187

## Data Availability

The original contributions presented in this study are included in the article. Further inquiries can be directed to the corresponding author.
